# Rod‐Shaped Nanotherapeutics Alleviate Rheumatoid Arthritis by Precisely Disrupting Platelet‐Mediated Pathological Crosstalk via a Morphology‐Dependent Manner

**DOI:** 10.1002/advs.202518005

**Published:** 2025-11-20

**Authors:** Bin Zhang, Yuanyi Hua, Xin Wen, Zhumei Liao, Jianheng Ren, Yajun Weng, Qin Wang

**Affiliations:** ^1^ Institute of Biomedical Engineering College of Medicine Southwest Jiaotong University Chengdu 610031 China

**Keywords:** cell interaction, platelet targeting, rheumatoid arthritis, rod‐shaped nanoparticles

## Abstract

During flares of rheumatoid arthritis (RA), activated platelets (PLTs) amplify inflammatory cascades and drive disease progression by extensively engaging in pro‐inflammatory crosstalk with inflammatory cells. Precisely disrupting PLT‐mediated pathological cellular crosstalk has emerged as promising therapeutics. Existing PLT‐targeting strategies primarily rely on the ligand‐mediated recognition, overlooking the hemodynamic behavior of circulating PLTs. Under blood flow, PLTs preferentially roll along vascular walls, while conventional nanocarriers tend to flow along the central axis. This inherent spatial separation limits their targeting efficiency. The optimization of nanoparticle geometry provides a potential solution by enabling precise control over their flow trajectories in circulation. Herein, the study designs fucoidan‐functionalized nanoplatforms with different morphologies and comparatively explores their targeting efficiency and regulatory activity in PLTs. In a microfluidic flow model, rod‐shaped nanoparticles exhibit markedly enhanced co‐localization with endothelial‐adherent PLTs. Moreover, these rod‐shaped nanoparticles inhibit focal adhesion kinase (FAK) and PI3K/AKT signaling in a morphology‐dependent manner, thereby effectively disrupting their pathological cellular crosstalk. In arthritic rats, intravenously administered resveratrol‐loaded nanorod (PNR@Res) efficiently binds circulating PLTs and accumulates in inflamed joints, ultimately effectively alleviating RA symptoms. The findings offer insight into how nanoparticle geometry governs cell interactions with circulating PLTs and influences PLT pathological behaviors through a morphology‐dependent mechanism in inflammatory diseases.

## Graphical abstract

Owing to the margination effect, rod‐shaped nanoparticles exhibit markedly enhanced co‐localization with endothelial‐adherent platelets (PLTs) under dynamic blood flow. When internalized by PLTs, rod‐shaped PNR@Res inhibit FAK and PI3K/AKT signaling in a morphology‐dependent manner, thereby disrupting their pathological crosstalk with various inflammatory cells. After administration, PNR@Res efficiently binds circulating PLTs and accumulates in inflamed joints, ultimately effectively alleviating RA.

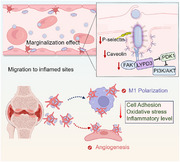

## 1 Introduction

Rheumatoid arthritis (RA) is a chronic autoimmune disorder in which a variety of pro‐inflammatory cell types, multiple inflammatory signaling pathway, and dysfunctional cytokines collectively form a complicated inflammatory network.^[^ ^1^, ^2^, ^3^ ^]^ Platelets (PLTs), representing the second most abundant cell type in peripheral blood, are traditionally recognized for their key role in hemostasis. However, emerging evidences now highlight their significant contribution to RA pathogenesis.^[^ ^4^, ^5^ ^]^ The number of circulating activated PLTs is extremely increased in RA patients and arthritic animal models.^[^ ^6^ ^]^ In RA condition, they continuously patrol the circulatory system, rapidly responding to inflammatory stimulus from damaged vessels and promptly migrating to inflamed synovium.^[^ ^7^ ^]^ The anucleate structure endows PLTs with morphological and behavioral flexibility, facilitating their unrestricted trafficking within inflamed tissues. PLTs possess a diverse array of membrane receptors, enabling their active crosstalk with various inflammatory cells.^[^ ^8^ ^]^ For example, activated PLTs express high level P‐selectin and release chemokines, including CXCL7 and CXCL4, greatly promoting the recruitment of inflammatory cells and cellular adhesion with neutrophils and monocytes.^[^ ^9^, ^10^ ^]^ In addition, activated PLTs can adhere to inflamed vascular endothelium and produce growth factors, thereby driving pannus formation.^[^ ^11^ ^]^ Moreover, PLTs contain abundant granules packed with biologically active compounds, and they would rapidly release these inflammatory mediators and amplify synovial inflammation after being recruited to inflamed sites.^[^ ^12^ ^]^

Due to the innate chemotaxis to inflamed sites, PLTs‐inspired drug carrier can achieve selective delivery to inflamed synovium. Currently, PLT‐mimicking strategies (e.g., PLT membranes or PLT‐derived vesicles coating) and engineered PLTs technologies have been widely adopted to achieve targeted delivery to pathological sites.^[^ ^13^, ^14^, ^15^ ^]^ Nevertheless, the complicated fabrication procedures, inconsistent batch‐to‐batch reproducibility, and the thrombotic risk associated with undesirable PLT activation during ex vivo processing hindered further application.^[^ ^16^ ^]^ Given the key pathological behavior of PLTs in RA pathogenesis, therapeutic strategies capable of precisely inhibiting PLT activation and their pathological crosstalk with inflammatory cells offer a promising direction for RA treatment.^[^ ^17^ ^]^ Therefore, in situ incorporating therapeutics into circulating PLTs offers a dual‐functional approach, combining the inflammation‐homing delivery of PLTs with therapeutic modulation of PLT‐mediated pathological cell crosstalk.

**Scheme 1:**
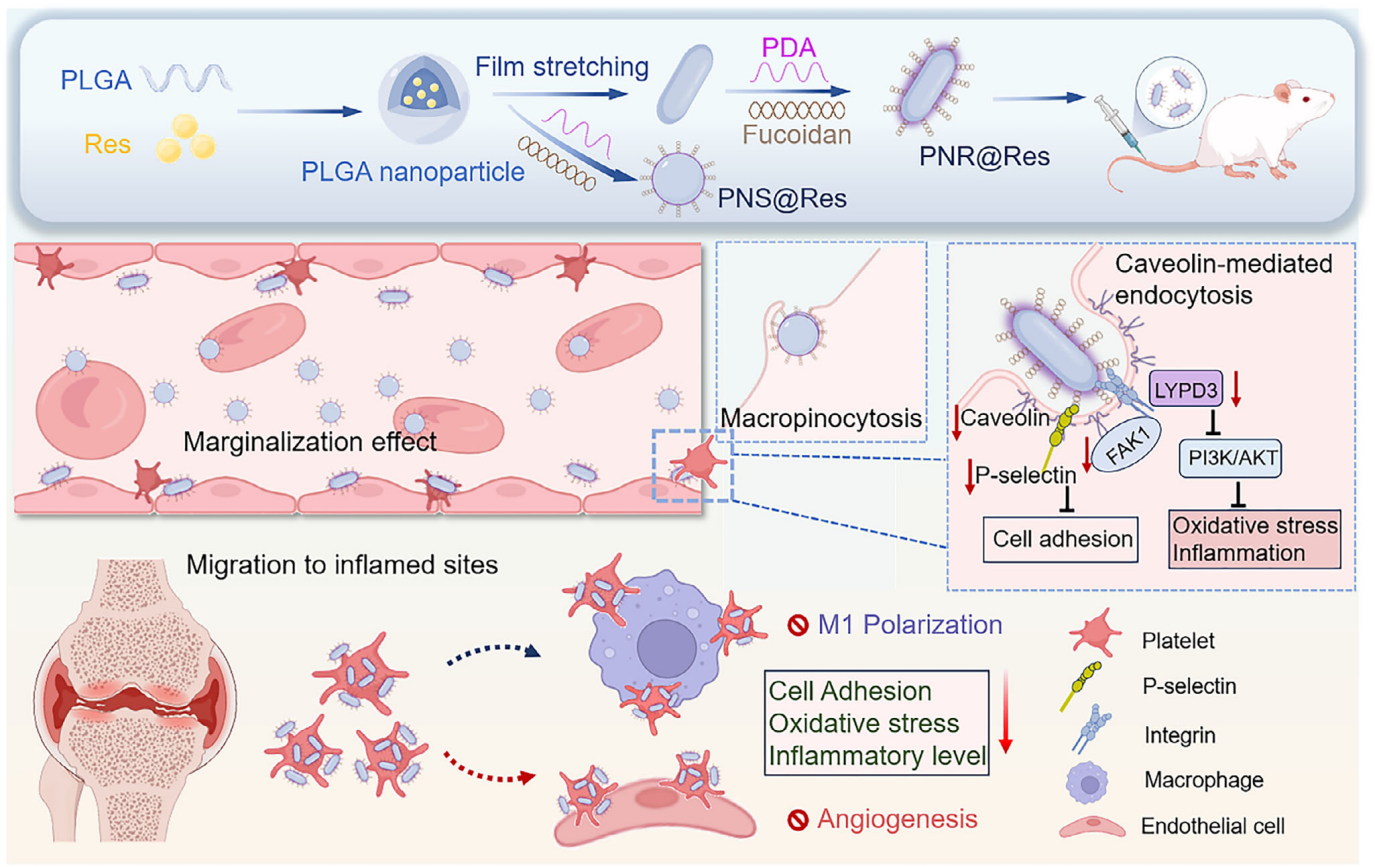
Schematic illustration of the preparation of PLT‐targeting nanoparticles with different morphologies, and their morphology‐dependent mechanism for precisely modulating PLT‐mediated pathological crosstalk with various inflammatory cells in RA treatment.

Existing PLT‐targeting strategies primarily involved surface modification of nanocarriers with fucoidan or PSN peptides, which could facilitate specific PLT binding via their high affinity to P‐selectin.^[^ ^18^, ^19^ ^]^ However, these ligands‐modified nanocarriers only considered PLT‐targeting requirements in static condition, yet failing to address the challenges of PLT targeting under dynamic blood flow. In dynamic blood flow, larger erythrocytes preferentially migrate toward the central axis of blood vessels under the shear force, forcing the smaller PLTs to move toward the vascular wall.^[^ ^20^ ^]^ The margination effect of PLTs makes them preferentially tumble and roll along vascular walls during blood flow, enabling rapid vascular surveillance and injury response.^[^ ^21^ ^]^ The fluid dynamics of nanoparticles in blood circulation are critically influenced by their geometry. Typically, spherical nanoparticles tend to flow toward the central axis, making them less likely to marginate toward vessel walls. In contrast, rod‐shaped nanoparticles tend to rotate and oscillate along vascular walls due to their anisotropic shape, leading to greater margination than nanospheres with similar size.^[^ ^22^ ^]^ Therefore, the unique flow dynamics of rod‐shaped nanoparticles are more likely to encounter the endothelial‐adherent PLTs. In addition to the greater propensity to encounter circulating PLTs, rod‐shaped nanoparticles generally show reduced phagocytosis by reticuloendothelial phagocytic system and prolonged retention in circulation.^[^ ^23^ ^]^ Based on this rationale, we designed fucoidan‐modified and resveratrol (Res)‐loaded poly (lactic‐co‐glycolic acid) (PLGA) nanoplatform with different morphologies (spherical PNS@Res and rod‐shaped PNR@Res), and comparatively explored their targeting efficiency and regulatory activity in activated PLTs (**Scheme** **1**). We employed a microfluidic chip to simulate dynamic blood flow and evaluated the uptake behaviors of spherical PNS@Res and rod‐shaped PNR@Res under mimic shear flow. Our study clearly elucidated how nanoparticle morphology influenced PLT targeting efficiency under hemodynamic condition, alongside the regulatory effect on PLT activation and inflammatory responses. Furthermore, we revealed the cellular interactions between drug formulation‐internalized PLTs with various inflammatory cells. In an arthritic model, we comparatively assessed the in vivo fate and anti‐RA therapeutic efficacy of PNS@Res and PNR@Res.

Here, we reported for the first time the superior PLT‐targeting capability of rod‐shaped PNR@Res over spherical nanoparticles in dynamic blood flow. Surprisingly, our findings revealed that unlike their spherical counterparts, these rod‐shaped PNR@Res effectively suppressed PLT activation and inhibited PLT‐inflammatory cell interaction by inhibiting focal adhesion kinase (FAK1) and PI3K/AKT signaling through a morphology‐dependent mechanism (Scheme 1). In arthritic rats, intravenously administered rod‐shaped PNR@Res efficiently bound circulating PLTs and utilized their intrinsic inflammatory tropism for subsequent trafficking to inflamed joints. In inflamed microenvironment, PNR@Res significantly disrupted the PLT‐mediated pathological crosstalk with various inflammatory cells. Moreover, the treatment of PNR@Res effectively reduced PLT‐mediated recruitment and infiltration of inflammatory cells, and ultimately alleviated RA symptoms.

## 2 Results

### 2.1 Preparation and Characterization of PNS and PNR

**Figure 1:**
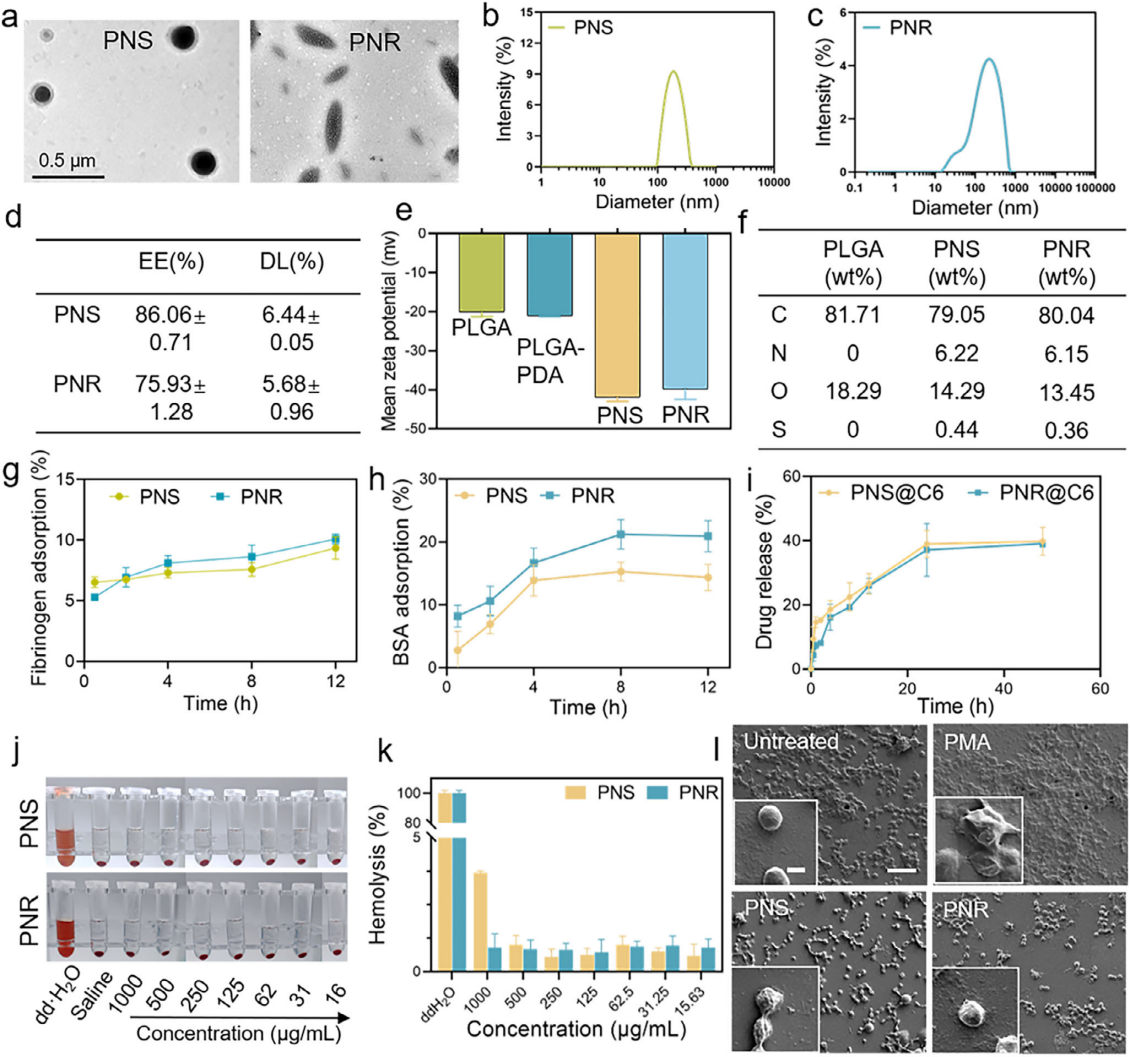
Characterization of PNS@Res and PNR@Res. a) TEM images of PNS and PNR. Size distribution of b) PNS and c) PNR. d) Res loading yield and encapsulation efficiency. e) Zeta potential of PLGA nanoparticles, PLGA‐PDA nanoparticles, PNS, and PNR. f) EDS analysis of PLGA nanoparticles, PNS, and PNR. g) Fibrinogen and h) BSA adsorption on PNS and PNR after incubation for 12 h. i) Drug accumulative release profile of PNS and PNR. j) Hemolysis images and k) hemolysis rate of PNS and PNR. l) The morphology changes and aggregation behaviors of PLTs after incubation with PNS and PNR, scale bar = 100 µm, inset scale bar = 2 µm. Results were presented as means ± SD, *n*= 3.

TEM images showed that the prepared PNS and PNR particles displayed typically spherical and rod‐shaped morphology as expected (**Figure** **1**a). The average aspect ratio of PNR was ≈2.69, which was calculated at the average ratio of the highest to the lowest dimension over dozens of nanorods in TEM images. DLS measurements indicated the average size of PNS and PNR were respectively 197.93 and 286.10 nm (Figure 1b,c). Due to the elongated shape, PNR showed a much wider diameter distribution than PNS. As shown in Figure 1d, PNS and PNR exhibited similar drug loading (DL) yield and encapsulation efficiency (EE), suggesting the film stretching process did not affect the drug loading. After fucoidan modification, the absolute value of zeta potential of PNS and PNR underwent a dramatic increase from −20 to ‐40 mV, indicating the successful coating of fucoidan shell (Figure 1e). In addition, the presence of N and S elements in the Energy dispersive X‐ray spectroscopy (EDS) analysis of PNS and PNR further confirmed the successful introduction of polydopamine (PDA) and fucoidan onto the particles (Figure 1f). Both PNS and PNR showed excellent colloidal stability in PBS and serum, with negligible variation in particle size, PDI, and turbidity within one week (Figure S1, Supporting Information).

Protein adsorption has been considered as the first event that rapidly occurs when a drug carrier enters the blood circulation. Meanwhile, moderate adsorption of albumin can partially reduce phagocytosis by macrophages and complement activation. In contrast, fibrinogen adsorption onto the nanocarriers may trigger PLT activation, increasing the risk of coagulation and thrombosis formation.^[^ ^24^ ^]^ When incubated with albumin and fibrinogen for a period, PNR displayed slightly higher albumin adsorption than PNS. However, both PNS and PNR showed very low fibrinogen adsorption, suggesting that they had minimal potential to induce PLT activation and coagulation (Figure 1g,h). In Figure 1i, PNS and PNR exhibited extremely similar drug release kinetics, with slow and sustained release profile. Cytotoxicity assessment indicated that both PNS and PNR possessed favorable biocompatibility, showing unaffected cell viability of RAW264.7 or HUVEC at all concentration ranges (Figure S2, Supporting Information). The hemolysis ratio of PNS and PNR was below 5 % even at the highest concentration (Figure 1j,k). In SEM images of PLT morphology, PNS and PNR‐treated PLTs showed no observable pseudopodia or filopodia formation compared with PMA‐treated group, suggesting good blood compatibility (Figure 1l).

### 2.2 The Uptake Efficiency and Mechanism of PNS and PNR in PLTs

**Figure 2:**
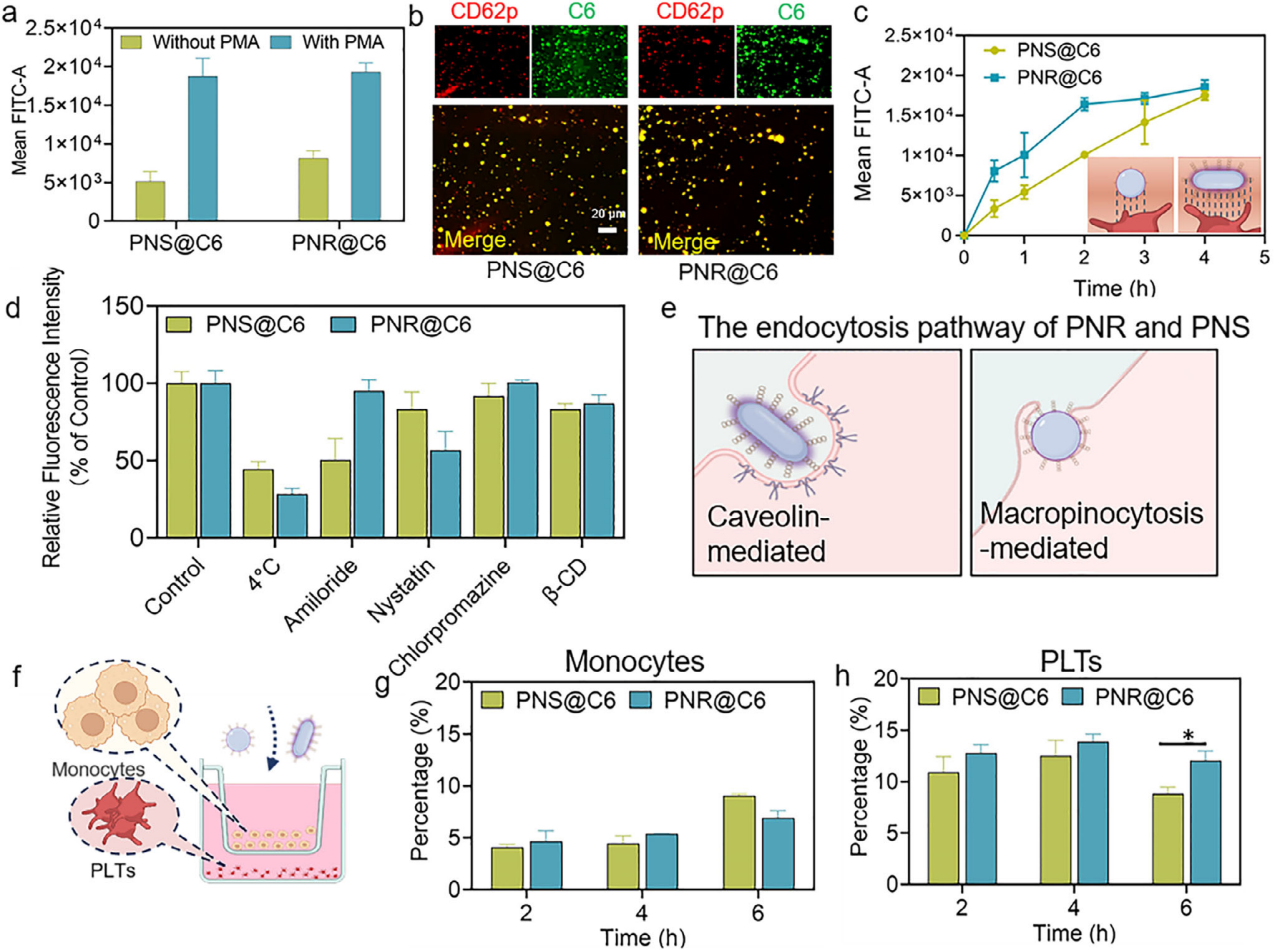
The uptake efficiency and mechanism of PNS and PNR in PLTs. a) Cellular uptake efficiency of PNS and PNR in PLTs with or without PMA stimulation. b) Fluorescent images of the co‐localization of activated PLTs with PNS and PNR, CD62p (red) indicated PLTs. c) The cellular uptake kinetics of PNS and PNR in PLTs. Inset indicates the binding areas of PNS and PNR with PLTs. d) The relative uptake efficiency of PNS and PNR in PLTs in the presence of various endocytosis inhibitors. e) Schematic illustration of the endocytosis pathway of PNS and PNR. f) The experiment design of competitive uptake behaviors of PNS and PNR between monocytes and PLTs. The comparison of uptake efficiency of PNS and PNR in g) monocytes and h) PLTs. Results were presented as means ± SD, *p < 0.05, *n*= 4.

When incubated with PLTs for 4 h, PNS and PNR showed comparable uptake efficiency both in resting and activated PLTs. Notably, activated PLTs displayed much higher uptake amount of PNS and PNR compared with resting PLTs (**Figure** **2**a). The enhanced uptake efficiency may result from the overexpression of P‐selectin on activated PLTs, which mediates the specific binding with fucoidan‐modified particles. The visualized comparison between PNS and PNR in terms of cellular uptake in activated PLTs is presented in Figure 2b. The fluorescent images showed extensive colocalization of PNS and PNR with PLTs. Since prolonged incubation time might contribute to the increased uptake efficiency observed for both PNS and PNR, we subsequently investigated the uptake kinetics to provide a detailed comparison of their internalization dynamics. The uptake kinetics were studied by incubating PNS or PNR with activated PLTs for different time spans. As shown in Figure 2c, PNR showed higher uptake efficiency than PNS in activated PLTs at initial timepoint. Specifically, the uptake amount of PNR was nearly 1.5‐fold higher than that of PNS in activated PLT at 2 h after incubation. The difference became less significant as the incubation time increased. These results demonstrated that PNR exhibited faster uptake kinetics in activated PLTs compared to PNS. Unlike spherical nanoparticles, elongate particles might potentially increase the propensity of nanoparticle‐cell contact, as illustrated in Figure 2c (inset). Therefore, the enhanced uptake kinetics of PNR might be attributed to the increased binding area and contact points with PLTs.

Cellular endocytic pathways of PNS and PNR in PLTs were studied in the presence of various endocytosis inhibitors, including β‐methyl cyclodextrin (β‐CD), amiloride, nystatin, and chlorpromazine (Figure 2d). The uptake of PNS was mainly through micropinocytosis process, while the uptake of PNR was primarily mediated via caveolin‐dependent endocytic pathway, as illustrated in Figure 2e. Although 4 °C treatment remarkably suppressed the endocytic process of PNS and PNR, it did not completely inhibit their uptake behavior, indicating that the binding of PNS and PNR to PLTs was not entirely energy‐dependent. Due to their nanoscale size, neither PNS nor PNR can passively diffuse into cells, implying that a portion of them may be adsorbed on the cell surface. We further accurately analyzed the intracellular PNS@C6 or PNR@C6 within PLTs. As shown in Figure S3a (Supporting Information), the intracellular content of PNS@C6 accounted for ≈56% of the total uptake amount, which was significantly lower than that of PNR@C6 (≈71%). Conversely, a considerable proportion of PNS@C6 was adsorbed on the cell surface, while the amount of PNR@C6 adsorbed on the cell surface was relatively low (Figure S3b, Supporting Information). Therefore, it can be concluded that despite similar overall cellular uptake level after 4 h of incubation with PLTs, a greater proportion of PNR actually entered the intracellular matrix compared to PNS.

In blood circulation, nanocarriers are likely to encounter abundant monocytes, which are responsible for phagocytosing and removing these exogenous nanocarriers. To compare the uptake efficiency of PNS and PNR in monocytes and PLTs, we established a transwell co‐culture system of monocytes and PLTs to simulate the physiological condition in circulation (Figure 2f). At all tested time points, the PLT population exhibited significantly higher uptake amount of PNS and PNR compared to monocytes (Figure 2g,h). This phenomenon may be attributed not only to the fucoidan‐mediated recognition, but also to the higher abundance of PLTs (≈10^11^/L) than monocytes (≈10^8^/L) in circulation. Notably, monocytes showed markedly lower uptake of PNR compared to PNS at 6 h. This difference likely came from the preferential phagocytosis of spherical nanoparticles over rod‐shaped nanoparticles by monocytes, which was consistent with findings reported in previous studies.^[^ ^25^ ^]^

### 2.3 The PLT‐Targeting Efficiency of PNS and PNR in Mimic Dynamic Blood Flow

**Figure 3:**
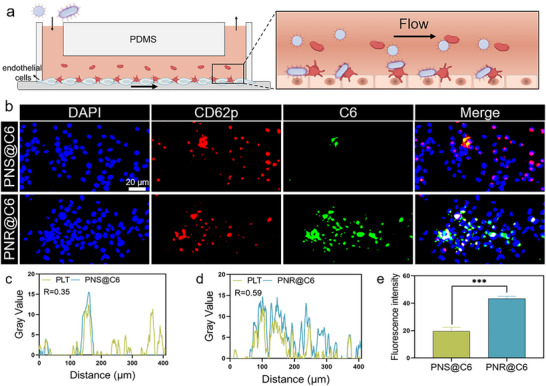
The PLT‐targeting efficiency of PNS and PNR in mimic dynamic blood flow. a) The mimic dynamic blood flow using a microfluidic chip with a monolayer of vascular endothelial cells at the bottom of the channel. b) The fluorescent co‐localization of PLTs with PNS or PNR after perfusion in microfluidic chip for 1 h. The quantitative analysis of colocalization between PLT (Red) and c) PNS or d) PNR. e) Semi‐quantification of the green fluorescence signal of PNS and PNR in the field. Results were presented as means ± SD, ***p < 0.001, *n*= 3.

Under blood circulation, when the shear force acts on the blood cells, erythrocytes undergo lateral migration in the central axis of blood vessels, resulting in the formation of a cell‐free layer near the vessel walls, where abundant PLTs are preferentially enriched. Various studies have confirmed the margination and adhesion behaviors of PLTs in vessel walls. Here, we aimed to systematically examine the uptake behavior of spherical and rod‐shaped nanoparticles by margination‐prone PLTs under simulated dynamic blood flow. As illustrated in **Figure** **3**a, we performed a microfluidic study to explore the co‐localization of PLTs and fluorescence‐labeled PNS or PNR. The bottom of the microfluid channel was seeded with a monolayer of vascular endothelial cells. After PLTs and nanoparticles (PNS or PNR) were perfused over the patch for 1 h, the uptake of PNS or PNR by PLTs was verified using a fluorescence microscope. As shown in Figure 3b, significant co‐localization of PNR (green fluorescence) with PLTs (red fluorescence) could be found along the vascular wall (blue fluorescence), with a markedly higher co‐localization coefficient compared to the PNS group (Figure 3c,d). Semi‐quantitative analysis of fluorescence intensity in Figure 3e revealed that the uptake of PNR in PLTs was much higher than that of PNS. As a result, margination‐prone PLTs more readily interacted with rod‐shaped PNR rolling along the vessel walls under dynamic flow condition, thereby exhibiting a distinct uptake preference for PNR over PNS.

### 2.4 The Effect of PNS@Res and PNR@Res on the Activation and Inflammatory Level of PLTs

**Figure 4:**
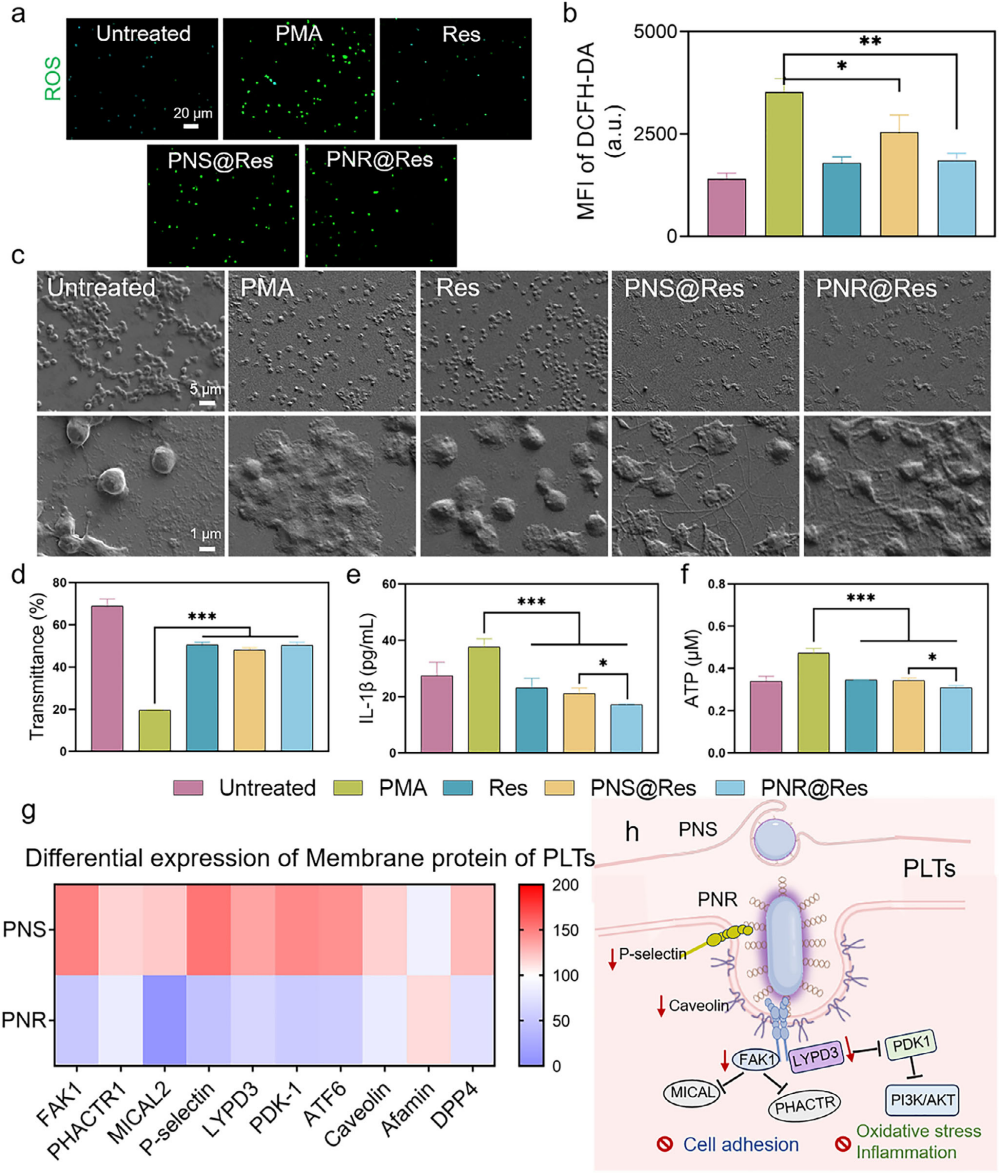
The effect of PNS@Res and PNR@Res on the activation and inflammatory level of PLTs. a) The fluorescent ROS images of PLTs after various treatments. b) The quantitative analysis of fluorescence intensity of ROS in PLTs after various treatments. c) The morphology changes of activated PLTs after various treatments observed using a SEM. d) The light transmittance of activated PLTs after various treatments measured using DLS. e) The supernatant level of IL‐1β and f) intracellular ATP within activated PLTs after various treatments. g) The proteomic analysis of differentially expressed membrane proteins of PLTs after PNS and PNR treatment. h) The potential regulatory mechanism and signaling pathway underlying the reduction in PLT activation and inflammation level induced by PNR treatment. Results were shown as mean ± SD, *p < 0.05, **p < 0.01, ***p < 0.001. *n*= 4.

When incubated with activated PLTs for 12 h, the effect of Res‐loaded PNS@Res and PNR@Res on PLT activation and inflammatory level were comprehensively assessed. Activated PLTs typically exhibit functional and morphological alterations, including elevated oxidative stress and inflammatory response, as well as extensive formation of pseudopodia and cell aggregation.^[^ ^26^ ^]^ As shown in **Figure** **4**a, we could observe that all treatment groups, including free Res, PNS@Res, and PNR@Res, effectively scavenged ROS level, indicating the potent antioxidative efficacy of Res. The quantitative analysis of ROS level clearly indicated PNR@Res possessed better ROS scavenging efficacy than PNS@Res (Figure 4b). Upon activation, PLTs exhibited significant aggregation accompanied by extensive pseudopodia and filament formation. While after incubation with various treatments, PLTs aggregation could be remarkably reduced. However, residual pseudopodia were still observed in both PNS@Res and PNR@Res‐treated PLTs (Figure 4c). The PLT aggregation can lead to a dramatic decrease in light transmittance.^[^ ^27^ ^]^ In Figure 4d, all treatment groups maintained the light transmittance of PLTs comparable to the normal group. These results indicated that both PNS@Res and PNR@Res can effectively suppress PLT activation and aggregation.

Activated PLTs could transform into key inflammatory players by generating excessive ATP and inflammatory cytokines.^[^ ^28^ ^]^ Quantitative analysis of intracellular ATP level and inflammatory cytokines revealed that all drug formulations significantly attenuated pathological ATP overproduction and IL‐1β release (Figure 4e,f), and PNR@Res demonstrated the most potent inhibitory effect, reducing ATP level to 52% and decreasing IL‐1β release by 43% of activated PLTs.

The abovementioned uptake study showed comparable uptake efficiency of PNS and PNR in activated PLTs. Nevertheless, the equivalent cellular internalization led to different regulatory effect in the pathological behaviors of PLTs. We inferred that spherical and rod‐shaped nanoparticles might cause differences in membrane deformation and receptor binding density during the endocytosis process, thus triggering divergent downstream events, including cytoskeletal reorganization and intracellular signaling. These differential events could consequently lead to varying degrees of PLT activation and inflammatory level. To elucidate how nanoparticle morphology influenced the physiological behaviors of PLTs, we compared the impact of PNS and PNR on the expression profiles of PLT membrane proteins using proteomic analysis. Over 40 differentially expressed membrane proteins were identified, and key components exhibiting significant alterations were listed in Figure 4g. Compared with PNS, PNR significantly reduced the levels of P‐selectin and caveolin in PLT membranes. Since fucoidan‐modified PNR can specifically bind to P‐selectin and enter the cell via caveolin‐mediated endocytosis, we speculated that PNR may guide the degradation of P‐selectin and caveolin through a lysosome‐mediated protein degradation pathway. Given that elevated P‐selectin and caveolin levels were positively correlated with PLT activation and inflammatory cascades,^[^ ^29^ ^]^ PNR‐triggered decrease of P‐selectin and caveolin was expected to attenuate PLT‐mediated pathological behaviors. Moreover, PNR treatment effectively downregulated the expression of focal adhesion kinase (FAK1) compared with PNS. Reduced FAK1, combined with downregulated MICAL and PHACTR protein, can collectively lead to the intracellular actin depolymerization, thereby diminishing cell migration and adhesion.^[^ ^30^, ^31^ ^]^ Besides, PNR also decreased the expression of LY6/PLAUR Domain Containing 3 (LYPD3) and phosphoinositide‐dependent protein kinase 1 (PDK1), two positive regulators of pro‐inflammatory PI3K/AKT signaling.^[^ ^32^ ^]^ Thus, the downregulation of LYPD3 and PDK1 can ultimately inhibit oxidative stress levels and inflammatory cascades. In addition, we also observed elevated expression of antioxidative afamin and reduced pro‐inflammatory DPP4 in PLT membranes in PNR‐treated group compared with that in PNS.^[^ ^33^, ^34^ ^]^ Taken together, PNR‐mediated downregulation of these key membrane proteins contributed to the inhibition of PLT activation and pro‐inflammatory effect, suggesting a morphology‐dependent regulatory mechanism (Figure 4h).

### 2.5 The Interaction of PNR@Res‐Treated PLTs with Inflammatory Cells

**Figure 5:**
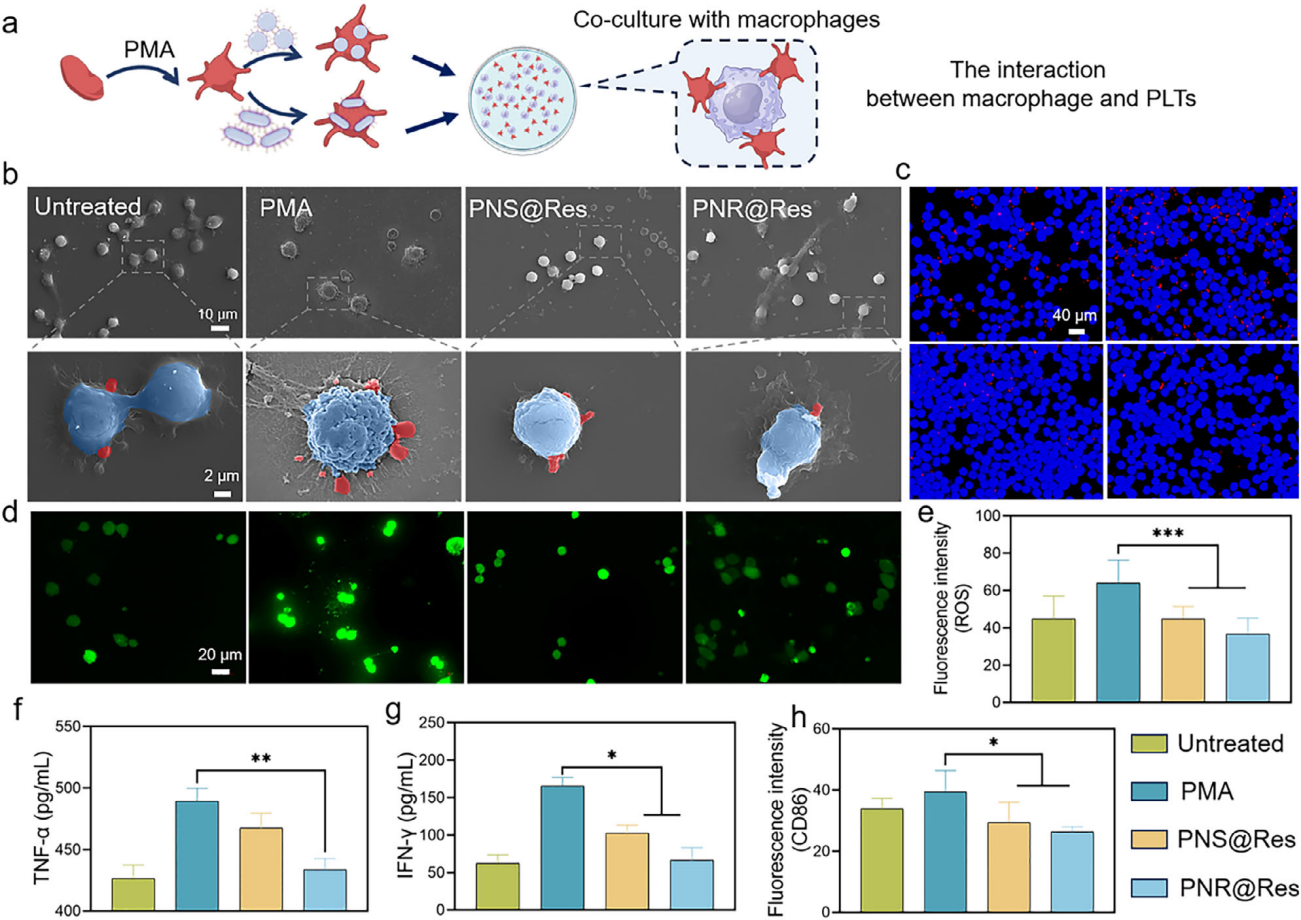
The interaction between PNR@Res‐treated PLTs and inflammatory macrophages. a) The experiment design for exploring the interaction between PNR@Res‐treated PLTs and inflammatory macrophages. b) SEM images of cell adhesion between PLTs (red) and inflammatory macrophages (blue). c) Fluorescence images of cell adhesion between PLTs (red) and macrophages (blue). d) Fluorescent ROS images and e) semi‐quantitative analysis of ROS intensity of macrophages after incubation with PNR@Res‐treated PLTs. The level of inflammatory cytokines, including f) TNF‐α and g) IFN‐γ produced by macrophages after incubation with PNR@Res‐treated PLTs. h) The fluorescence intensity of M1‐type specific marker CD86 in macrophages after incubation with PNR@Res‐treated PLTs. Results were shown as mean ± SD, *p < 0.05, **p < 0.01, ***p < 0.001. *n*= 4.

When circulating PLTs were massively recruited to inflamed sites, the abundant adhesion molecules in PLT membranes can facilitate the cellular crosstalk with diverse inflammation‐resident cells, including macrophages and vascular endothelial cells, ultimately amplifying inflammatory cascades and aggravating RA. These above‐mentioned results demonstrated PNR@Res‐treated PLTs exhibited significantly reduced inflammatory level. However, whether these PNR@Res‐treated PLTs can exert anti‐inflammatory effect when interacting with inflammatory cells remains to be elucidated. Here, we established a co‐culture system of PNR@Res‐treated PLTs and activated macrophages as illustrated in **Figure** **5**a. In SEM images, PNS@Res and PNR@Res‐treated PLTs displayed reduced adhesion to inflammatory macrophages compared with PMA‐activated PLTs (Figure 5b). Similarly, cell adhesion between PLTs and inflammatory macrophages was also explored using fluorescence co‐localization. In Figure 5c, activated PLTs displayed the highest co‐localization with macrophages, with extensive red fluorescence around macrophages. However, PNS@Res and PNR@Res‐treated PLTs exhibited very limited adhesion with activated macrophages, indicating that PNS@Res and PNR@Res treatment can minimize the inflammatory crosstalk between PLTs and macrophages. Moreover, both PNS@Res and PNR@Res‐treated PLTs significantly inhibited the production of ROS (Figure 5d,e) and inflammatory cytokines, including TNF‐α and IFN‐γ by macrophages (Figure 5f,g). Besides, these drug formulation‐treated PLTs effectively reduced the proportion of inflammatory M1 type macrophages (Figure 5h). It was evident that PNR@Res‐treated PLTs exhibited superior efficacy in suppressing the inflammatory level in macrophages compared to PNR@Res‐treated PLTs. As previously mentioned, PNR might reduce PLT adhesion and inflammatory levels in a shape‐dependent manner, thereby further attenuating the inflammatory response and disrupting the pathological cellular crosstalk.

**Figure 6:**
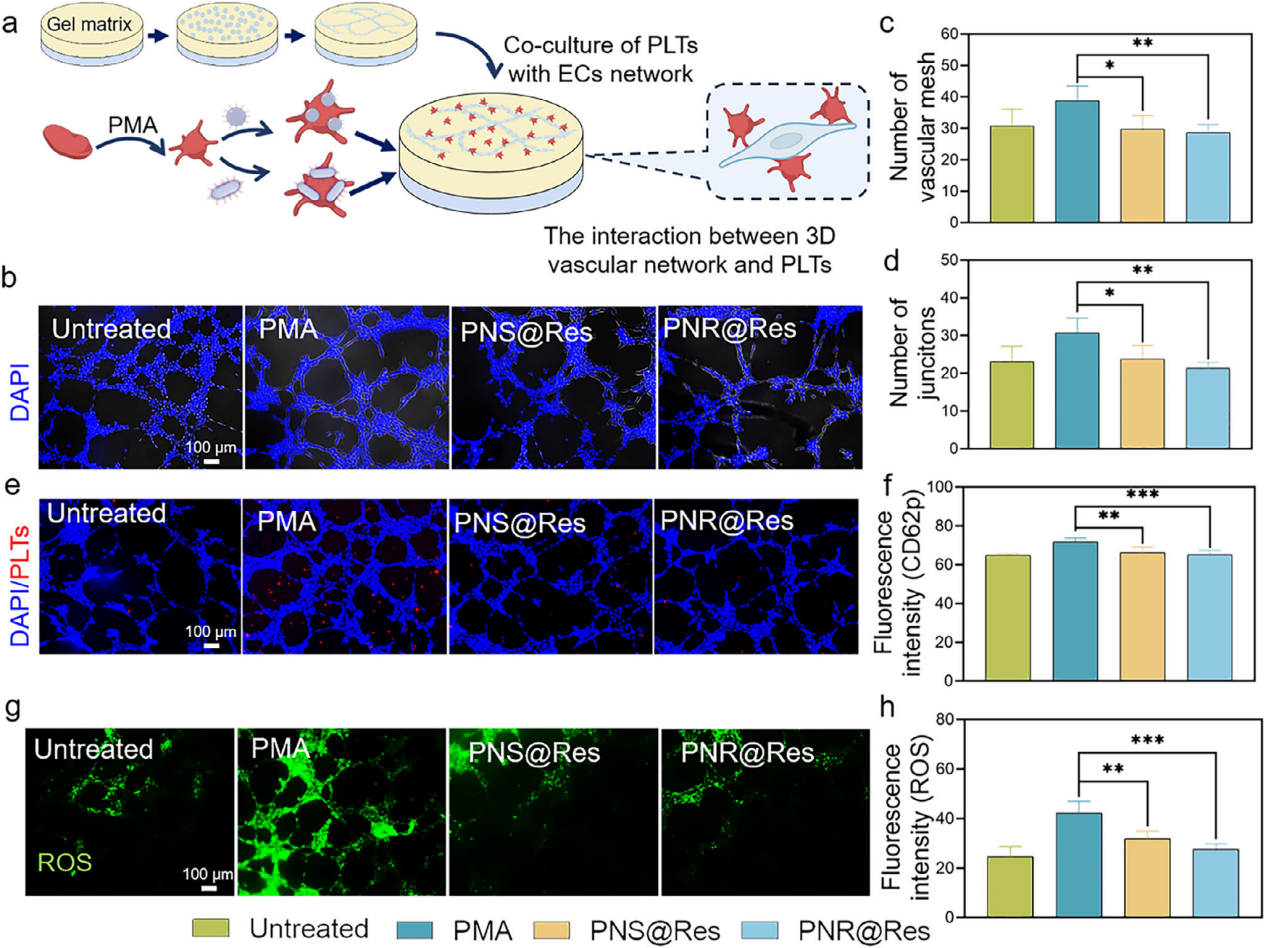
The interaction between PNR@Res‐treated PLTs and vascular networks. a) The experiment design for exploring the interaction between PNR@Res‐treated PLTs and 3D vascular network. b) The morphology of network structures formed by HUVEC after treatment of PNR@Res‐treated PLTs. Quantitative analysis of the numbers of c) vascular mesh and d) tube junctions of HUVEC network after treatment with PNR@Res‐treated PLTs. e) Cell adhesion between PNR@Res‐treated PLTs (Red) and HUVEC network (DAPI). f) Semi‐quantitative analysis of fluorescence intensity of PLTs adhered to the HUVEC network. g) Fluorescence ROS images and h) semi‐quantitative analysis of ROS intensity of HUVEC after incubation with PNR@Res‐treated PLTs. Results were shown as mean ± SD, *p < 0.05, **p < 0.01, ***p < 0.001. *n*= 4.

Within inflames synovium, activated PLTs often display firm adhesion to inflamed endothelium and engage in pathological crosstalk with endothelial cells, driving the dysregulated angiogenesis and pannus development. Here, we developed a biomimetic 3D vascular platform to systemically explore the interaction between PNR@Res‐treated PLTs with vascular network (**Figure** **6**a). As shown in Figure 6b, the complete vessel formed by endothelial cells could be clearly observed. The activated PLTs slightly promoted the tube formation. In contrast, PNS@Res and PNR@Res‐treated PLTs significantly interfered with the network formation. The quantitative analysis for capillary junction and vascular mesh provided in Figure 6c,d also confirmed this observation. These results indicated that activated PLTs could facilitate the pathological angiogenesis, while PNS@Res and PNR@Res‐treated PLTs could suppress the dysregulated angiogenesis and inhibit pannus formation. As shown in Figure 6e, activated PLTs exhibited extensive and firm adhesion to the vascular network. Conversely, drug formulation‐treated PLTs showed markedly decreased fluorescence co‐localization with endothelial cells. Particularly, PNR@Res‐treated PLTs displayed nearly undetectable red fluorescent signal within these tubular structures. Quantitative analysis of the fluorescent intensity of adherent PLTs further corroborated these findings (Figure 6f). Moreover, PNR@Res‐treated PLTs remarkably inhibited the ROS level in endothelial cells, as shown in Figure 6g, achieving nearly 30% reduction in ROS fluorescence compared with activated PLTs (Figure 6h). Similarly, compared to PNS, PNR@Res‐treated PLTs demonstrated superior performance in reducing cell adhesion and oxidative stress.

Collectively, upon encountering inflammation‐resident cells, these PNR@Res‐treated PLTs not only exhibited diminished cell adhesion, oxidative stress level, and inflammatory response, but also significantly suppressed M1 macrophage polarization and pathological neovascularization. Notably, comparative studies demonstrated the superior efficacy of PNR@Res‐treated PLTs over PNS@Res‐treated PLTs. These findings revealed that selective modulation of PLT activation and pathological behaviors in inflammatory milieu enabled precise regulation of diverse inflammation‐resident cells, thereby suppressing pro‐inflammatory cellular crosstalk and inflammatory cascade. These PNR@Res‐treated PLTs, armed with anti‐inflammatory Res and rod morphology, can serve as potent anti‐inflammatory modulators in the intricate inflammatory network.

### 2.6 The In Vivo PLT Targeting Efficiency and Biodistribution of PNS and PNR

**Figure 7:**
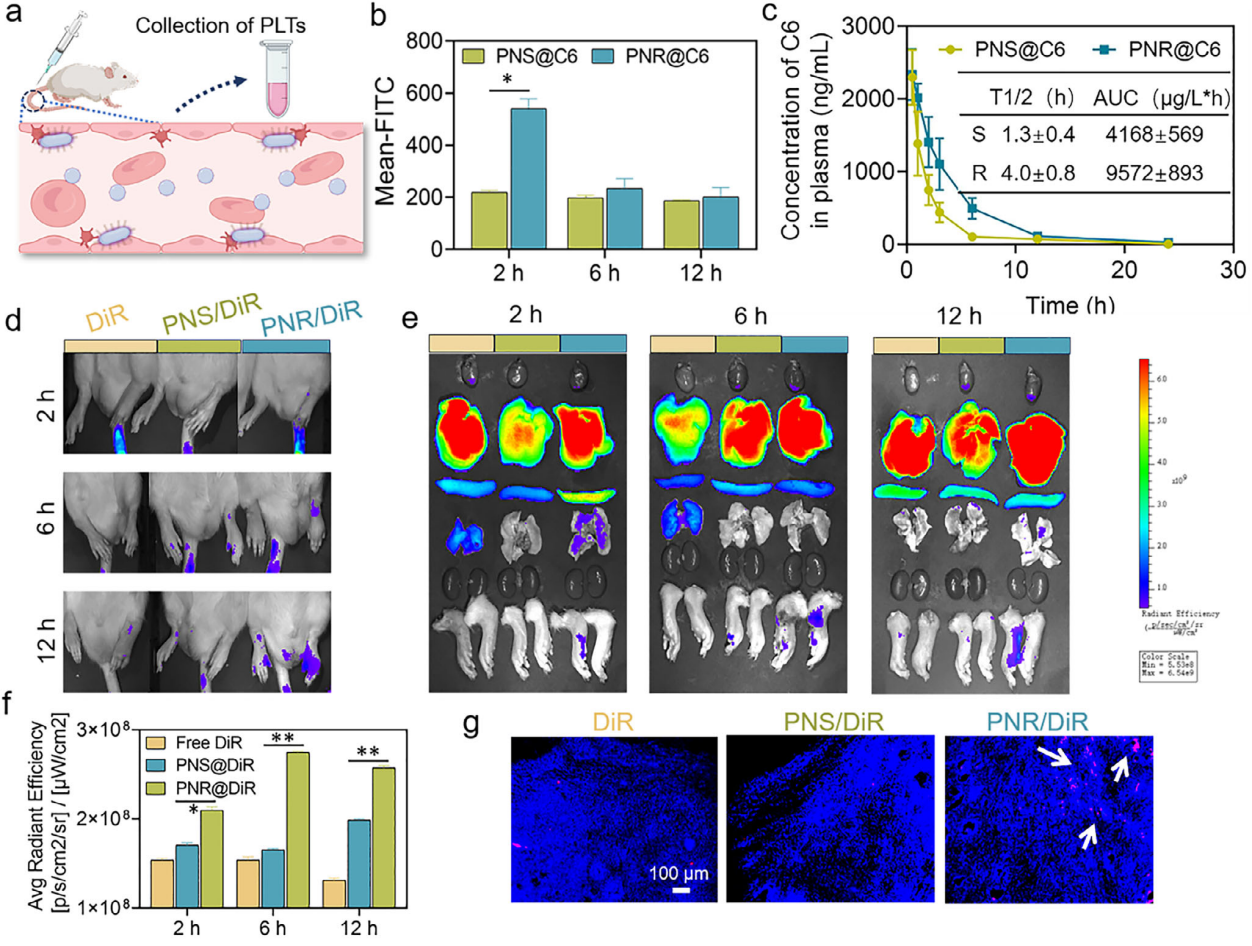
The in vivo PLT targeting efficiency and biodistribution. a)The experiment design for exploring the in vivo PLT targeting efficiency of PNS and PNR. b) The uptake efficiency of PNS and PNR by circulating PLTs at different time points. c) The pharmacokinetics profile of PNS and PNR after intravenous injection. Inset indicates the key pharmacokinetics parameters. d) The in vivo imaging of PNS and PNR distribution in arthritic rats. e) The ex vivo imaging of PNS and PNR distribution in arthritic joints and main organs (from up to down: heart, liver, spleen, lung, kidney, and joint). f) The semi‐quantitative analysis of fluorescence intensity of PNS and PNR in arthritic joints. g) Representative fluorescent images of PNS and PNR distribution in section of synovial tissues from AIA rats, white arrows indicated the red fluorescent areas. Results were shown as mean ± SD, *p < 0.05, **p < 0.01, ***p < 0.001. *n*= 4.

After intravenous administration, the in vivo PLT targeting efficiency of PNS and PNR was determined as shown in **Figure** **7**a. Figure 7b shows the in vivo uptake kinetics profile of PNS and PNR in circulating PLTs. At 2 h post‐injection, PNR achieved nearly 2.3‐times higher PLT targeting efficiency than PNS. The difference in uptake amount between PNS and PNR gradually diminished over time. Comparative pharmacokinetics profiles of PNS and PNR are presented in Figure 7c. Notably, PNR demonstrated significantly improved pharmacokinetics behaviors relative to PNS, exhibiting a 3.1‐fold prolonged half‐life in circulation and 2.3‐fold higher increase in AUC. The extended half‐life and bioavailability of PNR might result from the rod morphology, which facilitated the margination behavior in circulation and evaded macrophage phagocytosis.

The in vivo and ex vivo imaging of PNS and PNR in AIA rats showed that the distribution of PNR in arthritic joints was much higher than PNS at all tested time points, and exhibited more prolonged fluorescence retention compared to PNS (Figure 7d,e). Especially at 6 h post‐injection, PNR displayed approximately 2.2‐times higher accumulation in arthritic joints than PNS. This finding was further corroborated by semi‐quantitative analysis of fluorescence intensity in arthritic joints (Figure 7f). Comparative analysis of fluorescence sections further demonstrated that, at 24 h post‐injection, the PNR‐treated group maintained obvious fluorescence signal in joint tissues (white arrows), whereas only weak signal was detected in the PNS‐treated group, further validating the improved accumulation and prolonged retention of PNR in arthritic joints (Figure 7g). We speculated that the enhanced accumulation and retention in inflamed sites observed in PNR group may be attributed to its superior PLT‐targeting capability in vivo. Owing to the inherent inflammatory tropism, circulating PLTs engulfed PNR and subsequently transported them to the inflamed tissues. Apart from that, the long circulation performance of rod‐shaped PNR could synergistically contribute to the preferential distribution in arthritic sites.

### 2.7 The Therapeutic Efficacy in AIA Rats

**Figure 8:**
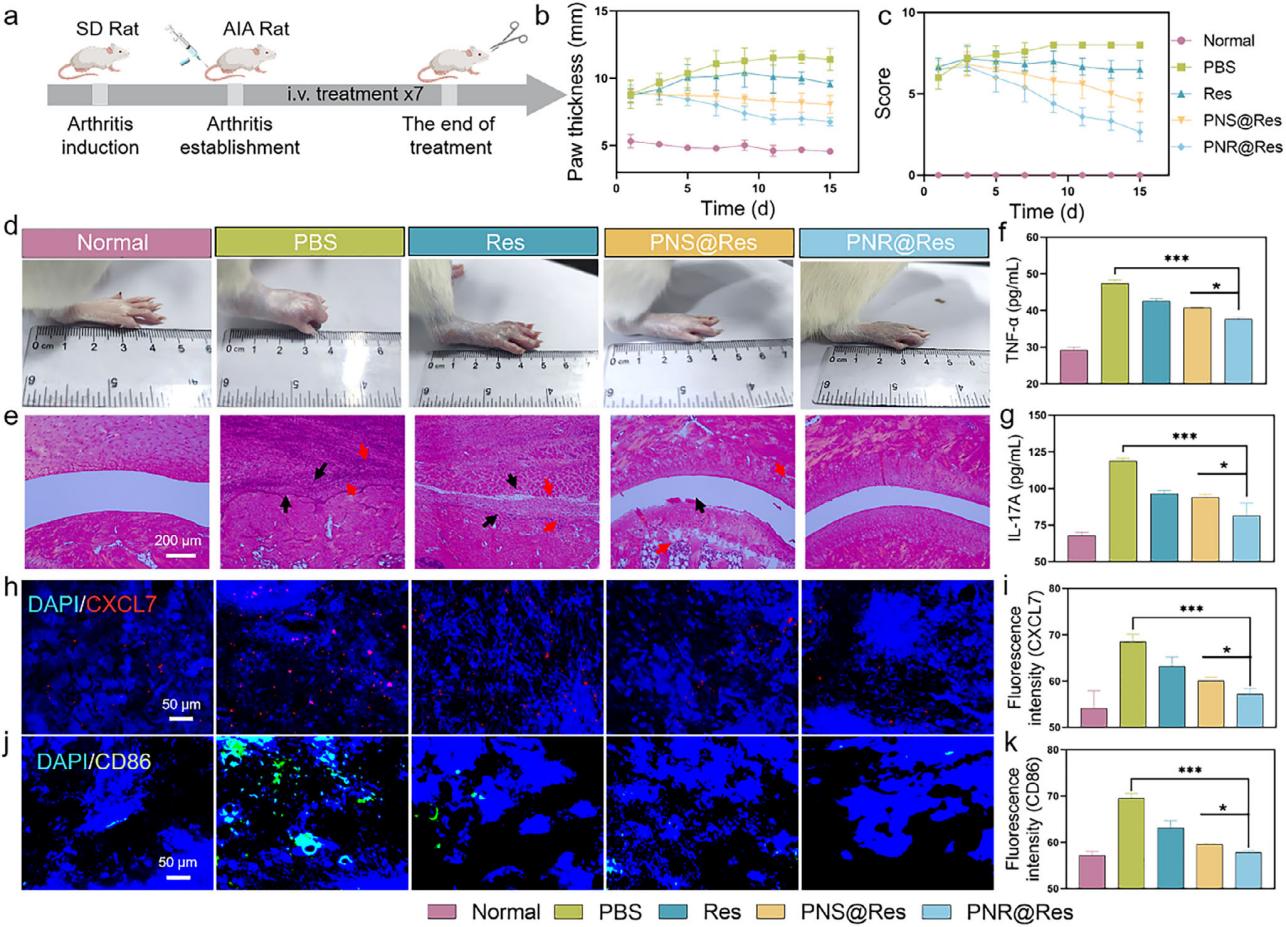
The therapeutic efficacy in AIA rats. a) The experiment design for evaluating the therapeutic efficacy of PNR@Res in AIA rats. The changes in b) paw thickness and c) clinical scores of AIA rats during the treatment process. d) Representative images of arthritic joints after various treatments. e) HE section of ankle joints from AIA rats receiving various treatments, black and red arrows indicated inflammatory infiltration and tissue damage, respectively. The level of inflammatory cytokines, including f) TNF‐α and g) IL‐17A, in arthritic joints of AIA rats after various treatments. Immunofluorescence staining of h) CXCL7 and j) CD86 in the section of inflamed synovium from AIA rats after various treatments. The semi‐quantitative analysis of fluorescence intensity of (i) CXCL7 and (k) CD86 in the section of the inflamed synovium. Results were shown as mean ± SD, *p < 0.05, **p < 0.01, ***p < 0.001. *n*= 6.

The therapeutic efficacy of PNS@Res and PNR@Res was explored in AIA rats, as shown in **Figure** **8**a. AIA rats with comparable joint swelling severity were enrolled for pharmacodynamics assessment. Figure 8b,c presented the change in paw thickness and clinical score of AIA rats during the treatment process, serving as direct parameters for evaluating joint swelling. Res treatment showed relatively limited efficacy in decreasing the paw thickness and score. In contrast, PNR@Res exhibited much higher efficacy in reducing paw thickness and score compared with PNS@Res and Res, yielding the most significant reduction in joint swelling among all groups. Photographic records of joint appearance further corroborated these findings (Figure 8d). The histological analysis of ankle joints indicated minimal inflammatory infiltration and cartilage erosion in PNR@Res group (Figure 8e), whereas in Res and PNS@Res treated groups, the inflammatory cells (black arrows) and tissue damage (red arrows) remained evident. The level of inflammatory cytokines in the arthritic joint is presented in Figure 8f,g. Compared with other treatment groups, PNR@Res treatment displayed the lowest expression of TNF‐α and IL‐17A, suggesting the superior anti‐inflammatory efficacy. The inflammation‐resident PLTs often largely generate chemokines to recruit and activate various immune cells, amplifying inflammatory cascades and aggravating tissue damage. The PLT‐derived CXCL7 has been considered as a key player in the recruitment of inflammatory cells.^[^ ^35^ ^]^ The immunofluorescent staining of ankle joints showed an extensive fluorescent signal of CXCL7 in PBS group. Both PNS@Res and PNR@Res effectively suppressed the CXCL7 level (Figure 8h,i), with PNR@Res demonstrating a more pronounced effect. These results indicated the potent efficacy of PNR@Res in suppressing the PLT‐mediated inflammatory infiltration. M1 type macrophages in inflamed synovium were the main producers of inflammatory cytokines. As shown in Figure 8j,k, PNR@Res significantly reduced the proportion of M1 type macrophages in contrast to free Res and PNS@Res treatment. Therefore, these results consistently demonstrated the superior therapeutic index of PNR@Res treatment, which greatly suppressed joint swelling, inflammatory response, and cartilage destruction in AIA rats by precisely modulating PLT‐mediated pro‐inflammatory behaviors. In addition, our treatment did not cause any obvious weight loss in all AIA rats (Figure S3a, Supporting Information). At the end of treatment, blood cell counts, including RBC, WBC, and PLTs of PNR@Res‐treated AIA rats, were all within normal range (Figure S3b–d, Supporting Information). In addition, PNR@Res did not lead to any abnormality in the serum level of AST, ALT, or BUN, indicating favorable in vivo biocompatibility. We explored the in vivo risk of coagulation after PNS@Res and PNR@Res treatments by monitoring the four coagulation indicators, including prothrombin time (PT), activated partial thromboplastin time (APTT), thrombin time (TT), and fibrinogen (FIB). As shown in Figure S5 (Supporting Information), both PNS@Res and PNR@Res treatments did not obviously impair the coagulation function, with all coagulation indicators falling within the normal range.

Taken together, our results confirmed the rod‐shaped PNR@Res could more efficiently target circulating PLTs than their spherical counterparts, due to their shared margination behavior and similar trajectories in dynamic blood flow. After phagocytosing these rod‐shaped PNR@Res, circulating PLTs can be efficiently recruited to inflamed joints, thereby promoting the targeted drug delivery and prolonged retention at inflammatory sites. More importantly, compared to conventional spherical nanoparticles, rod‐shaped nanoparticles inhibited FAK1 and PI3K signaling in a morphology‐dependent manner, thereby suppressing the pro‐inflammatory behavior of PLTs. Our study not only revealed the advantages and preference of rod‐shaped nanoparticles in PLT targeting due to their unique hemodynamic behavior, but also elucidated their morphology‐dependent regulatory mechanism on the pathological behavior of PLTs.

## 3 Discussion

After intravenous administration, rod‐shaped PNR were more readily deposited and reoriented on the blood vessel walls compared to spherical PNS, facilitating increased contact with PLTs adhering to the vessel walls. When interacted with PLTs, the low curvature of rod‐shaped PNR enabled a larger contact area and more binding sites, which promoted rapid cell binding and subsequent internalization. Our study revealed for the first time that rod‐shaped nanoparticles exhibited superior efficacy over their spherical counterparts in PLTs targeting under dynamic blood flow.

Our results indicated that spherical PNS were internalized more efficiently by macrophages than rod‐shaped PNR. We speculated that this difference can be attributed to the lower aspect ratio of spherical PNS, which would require less actin remodeling for cell internalization. Previous research conducted by Mitragotri and co‐workers validated that macrophages could not sufficiently internalize elongated particles. Thus, rod‐shaped particles often display prolonged in vivo circulation time due to the reduced endocytosis by macrophages. Compared with macrophages, PLTs generally have higher mobility and morphological flexibility due to the anucleate structure. As a result, the increased motility and morphological flexibility of PLTs might translate to their lower energy barrier for actin remodeling and phagocytosis of the elongated particles, compared with macrophages. Based on the rationale, we found macrophages appeared to preferentially take up spherical nanoparticles, while PLTs tended to internalize rod‐shaped particles.

During endocytosis process, rod‐shaped PNR led to the co‐transport of more P‐selectin and caveolin into lysosomes, triggering enhanced degradation of P‐selectin and caveolin in lysosomes. In contrast, spherical PNS are internalized primarily via macropinocytosis, which tends to induce more pronounced actin cytoskeleton remodeling. Consequently, compared with their spherical counterparts, rod‐shaped PNR decreased cytoskeleton remodeling‐driven inflammatory signaling in a morphology‐dependent manner, thereby further attenuating the pathological cellular crosstalk with other inflammatory cells. We speculated that these PNR@Res‐treated PLTs may inhibit the pathological interaction with inflammatory cells and exert immunomodulatory effects through several possible mechanisms: 1) These drug formulation‐incorporated PLTs can largely secrete drug‐containing vesicles, which are able to regulate macrophage polarization and inflammatory signaling after being internalized. 2) These engineered PLTs can also reduce the release of pro‐inflammatory mediators and generate anti‐inflammatory or immunosuppressive molecules (such as TGF‐β) under the action of Res, ultimately suppressing cellular adhesion and abnormal activation. 3) PNR@Res treatment downregulated the expression of important membrane receptors on the PLT surface (such as P‐selectin), thereby reducing pathological adhesion and crosstalk with inflammatory cells.

On the other hand, rod‐shaped PNR could more effectively resist the phagocytosis by reticuloendothelial phagocytic system and prolong retention in circulation compared with spherical PNS. Owing to the improved pharmacokinetics performance of PNR and intrinsic inflammatory tropism of PLTs, these PLT‐targeted PNR can efficiently accumulate in inflamed joints, ultimately exerting improved anti‐inflammatory efficacy. Collectively, the rod morphology of PNR not only facilitated its binding to PLTs and subsequent immunomodulatory effect, but also promoted prolonged circulation and targeted distribution to inflammatory sites.

## 4 Conclusion

In this study, we comparatively investigated the PLT targeting efficiency and mechanisms of nanoparticles with rationally designed morphologies. We found that rod‐shaped PNR@Res could more efficiently target circulating PLTs than their spherical counterparts, due to their shared margination behavior and similar trajectories in dynamic blood flow. Moreover, our findings revealed that, unlike their spherical counterparts, rod‐shaped PNR@Res effectively suppressed PLT activation and inhibited PLT‐inflammatory cell interaction through a morphology‐dependent mechanism. In arthritic rats, intravenously administered PNR@Res efficiently bound circulating PLTs and selectively accumulated in inflamed joints, where PNR@Res significantly suppressed pathological PLTs activation and disrupted their crosstalk with inflammatory cells, thereby effectively alleviating RA symptoms. Our study not only provided an efficient PLT‐targeted strategy for RA treatment, but also highlighted the critical role of morphology design in enhancing nanoparticle‐cell interaction and modulating pathological behaviors in inflammatory disorders.

## 5 Experimental Section

### Materials

Poly (lactic‐co‐glycolic acid) (PLGA50:50, *M*_w_:10 kDa) was provided by Evonik (Germany). Fucoidan, poly(vinyl alcohol) (PVA) 1788, and dopamine hydrochloride were supplied by Aladdin (Shanghai, China). Resveratrol (Res) and Couramin‐6 (C6) were provided by Meilunbio (Dalian, China). Antibodies, including anti‐CD62p, anti‐CD86, and anti‐CXCL7 were purchased from Thermo Fisher (MA, USA). ATP Detection Kit, ROS probe, and Matrix Gel were provided by Beyotime (Shanghai, China). TNF‐α, IL‐1β, and IL‐17A ELISA Detection Kits were from Biolegend (CA, USA). Male Sprague‐Dawley (SD) rats weighing ≈250 g were from Dashuo (Chengdu, China). Complete Freund's adjuvant (CFA) was purchased from Chondrex (Washington DC, USA). All animal experiments were approved by the Ethics Committee of Southwest Jiaotong University. The ethics approval number was SWJTU‐25010‐KJT (211).

### Preparation of PNS@Res and PNR@Res

PLGA (10 mg) and Res (3 mg) were dissolved in 1 mL of acetone, and the solution was gradually added to 2 mL of 1% (w/v) PVA solution. Acetone was removed at 42 °C using a rotary evaporator, and the mixture was centrifuged at 16 000 g for 1 h to collect drug‐loaded PLGA nanospheres. The prepared PLGA nanospheres were dispersed in 10% PVA solution, poured into a mold, and dried at 45 °C for 12 h to allow the film formation.^[^ ^36^ ^]^ After being fixed on an X‐axis manual displacement platform, the dried film was heated at 70 °C for 1 h and stretched. The stretched film was cooled at 4 °C for 20 min, followed by dissolving it in deionized water, and PLGA nanorods were collected by centrifugation. The obtained drug‐loaded PLGA nanospheres or nanorods were dispersed in 1 mL of deionized water, followed by the addition of 10 mg fucoidan and 1 mL of 1% (w/v) dopamine hydrochloride solution. The mixture was stirred for 30 min and then centrifuged at 16 000 g for 1 h to collect fucoidan‐modified drug‐loaded PLGA nanospheres (PNR@Res) or nanorods (PNS@Res).

### Characterization of PNS@Res and PNR@Res

Transmission electron microscope (TEM, JEOL TEM‐1011, Japan) was employed to observe the particle size and morphology of PNR and PNS. Elemental analysis was conducted using OXFROD X‐Max 80 to comparatively analyze the elemental composition of PNS and PNR after fucoidan modification. The hydrodynamic diameter, polydispersity index (PDI), and zeta potential were measured using dynamic light scattering (DLS, Zeta sizer Nano instrument, Anton Paar, Austria). For drug loading assessment, 1 mg PNS@Res or PNR@Res was dispersed in 1 mL of deionized water, and 4 mL of methanol was added. The mixture was subjected to ultrasonic treatment. Afterward, the solution was centrifuged at 16 000 g, and the drug content in the supernatant was measured using a high‐performance liquid chromatography (HPLC, Agilent 1260, USA).

### Stability Analysis

The hydrodynamic diameter and PDI of PNS and PNR (1 mg mL^−1^) at 4 °C were monitored for 7 consecutive days using DLS. For serum stability analysis, PNS and PNR (1 mg mL^−1^) were respectively mixed with an equal volume of rat serum. The optical density (OD) at 600 nm (termed as turbidity) was measured using a microplate reader (Bio‐Tek, MQX‐200, USA). The protein adsorption of PNS and PNR was investigated by incubating samples with bovine serum albumin (BSA) or fibrinogen (1 mg mL^−1^) at 37 °C at the shaking speed of 80 rpm. At indicated time points, samples were centrifuged at 16 000 g for 10 min, and the protein adsorption was measured and calculated using a microplate reader.

### In Vitro Release

PNS@C6 and PNR@C6 were respectively dispersed in 1 mL of PBS containing 0.1% Tween 80 and placed in dialysis bags. All samples were immersed in 10 mL of pH 7.4 PBS containing 0.1% Tween 80 at 37 °C. At predetermined time points, 100 µL of samples were withdrawn and fresh PBS was replenished. The fluorescence intensity in each sample was measured using a fluorescence spectrophotometer, and the accumulated drug release profile was calculated.

### Cytocompatibility

RAW264.7 and HUVEC cells were seeded in 96‐well plates at a density of 10^4^ cells/mL and cultured for 12 h. The cytotoxicity of PNS and PNR in two cells was assessed using the MTT assay. To compare the hemolytic rate of PNS and PNR, 1 mL of PNS and PNR at different concentrations was prepared and incubated with 200 µL of fresh rat blood at 37 °C for 1 h. Samples were centrifuged at 800 g for 10 min. The absorbance of the supernatant was measured at 545 nm, and hemolysis rate was calculated.

### PRP Collection and PLTs Activation

Fresh whole blood was collected from rats, and platelet‐rich plasma (PRP) was collected by centrifuging blood samples at 300 g for 10 min. 1 mL PNS and PNR (1 mg mL^−1^) were respectively mixed with 100 µL PRP and incubated for 4 h at 37 °C. PRP incubated with phorbol 12‐myristate 13‐acetate (PMA) at 80 nm was used as a positive control. The solutions were then dropped on the sterile silicon wafers and fixed using 4% paraformaldehyde. PLTs were dehydrated using a graded series ethanol, and the morphology of PLTs was captured using a scanning electron microscope (SEM, JSM‐7500F, JEOL, Japan).

### Cellular Uptake Efficiency and Mechanism

200 µL of PRP was seeded in a 48‐well plate, and 200 µL of culture media was added to each well. PMA at 80 nm was added to activate PLTs. For accurate analysis of cellular uptake, fluorescent dye C6 was incorporated into PNS and PNR. PNS@C6 and PNR@C6 at the final C6 concentration of 2 µg mL^−1^ were respectively incubated with PLTs at 37 °C for 4 h. After that, PLTs were resuspended and centrifuged at 1500 g for 20 min. The obtained PLTs pellets were analyzed using a flow cytometry (BD Accuri C6 Plus, USA). In parallel, PLTs were labelled using CD62p antibody, and the co‐localization of PLTs and nanoparticles was captured using a fluorescence microscope (ZEISS Axio Observer). The uptake kinetics of PNS and PNR in activated PLTs were studied by monitoring the uptake efficiency at indicated time points using a flow cytometry.

To investigate the endocytic pathways of PNS and PNR in PLTs, activated PLTs were seeded in a culture plate at the density of 10^5^ cells per well. Various inhibitors, including amiloride (30 µg mL^−1^), chlorpromazine hydrochloride (10 µg mL^−1^), nystatin (25 µg mL^−1^), or β‐cyclodextrin (10 µg mL^−1^), were added to each group and incubated with PLTs for 2 h to block certain endocytic pathways.^[^ ^37^ ^]^ PNS@C6 and PNR@C6 at the final C6 concentration of 2 µg mL^−1^ were subsequently added and incubated at 37 °C for further 4 h. After incubation, PLTs were collected and centrifuged at 1500 g for 20 min. Cell pellets were resuspended in PBS and analyzed using a flow cytometry.

PLTs and monocytes RAW264.7 were respectively inoculated into the lower chamber and insert of a transwell culture plate. PNS@C6 and PNR@C6 at the final C6 concentration of 2 µg mL^−1^ were added to the plate and incubated for a period. PMA at 80 nm was added to the co‐culture system to stimulate the inflammatory state. At the indicated time points, both cells were collected and lysed. After cell lysis, the supernatant was collected by centrifugation, and the intracellular fluorescence intensity was measured using a fluorescence spectrophotometer.

### Microfluidic Model of Blood Flow

HUVEC (10^7^cells/mL) were perfused into the microfluidic chip and incubated for 12 h to allow for sufficient cell adherence on the bottom. PLTs (10^9^cells/mL) were resuspended using culture medium containing PNS@C6 or PNR@C6 at the final C6 concentration of 2 µg mL^−1^. The mixture was injected into the microfluidic chip at a flow rate of 30 µL min^−1^ and maintained for 1 h.^[^ ^38^ ^]^ Non‐adherent PLTs and unbound nanoparticles were washed using PBS. After being fixed by 4% paraformaldehyde, HUVEC and PLTs were respectively stained with DAPI and anti‐CD62p antibody. The uptake of PNS@C6 or PNR@C6 by PLTs was observed using a fluorescence microscope.

### PLTs Activation

To compare the anti‐PLT activation efficacy of PNS@Res and PNR@Res, PLTs seeded in 24‐well plate were incubated with PNS@Res or PNR@Res at the Res concentration of 20 µM in the presence of PMA (80 nM). Cell cultures were incubated at 37 °C for 24 h. The supernatant of cell cultures was collected, and the level of inflammatory cytokines IL‐1β in supernatant was detected using a commercially available ELISA kit. The light transmittance of PLTs after treatments was measured using DLS. Intracellular ATP level of PLTs was measured via an ATP detection kit. After treatment, ROS probe DCFH‐DA (10 µm) was added and incubated with PLTs for 30 min. The intracellular ROS in PLTs was visualized using a fluorescence microscope. The morphology and aggregation of PLTs after treatment were observed by SEM. As for the proteomic analysis of membrane proteins of PLTs after PNS and PNR treatment, PLTs (10^9^ cells/mL) were incubated with PNS or PNR at final concentration of 100 µg mL^−1^ for 18 h. Subsequently, PLTs were lysed, and cell membranes were collected via ultracentrifugation. The obtained protein samples were further lysed with lysis buffer (containing 4 % SDS, 0.1 mol L^−1^ Tris HCl and DTT) and digested by trypsin to collect protein segments, which were then analyzed via a nano‐liquid chromatography tandem mass spectrometry (nanoLC‐MS/MS, Vanquish Bio‐Q Exactive Plus, Thermo Scientific, USA).

### The Interaction of PNR@Res‐Treated PLTs with Macrophages

PLTs seeded in 24‐well plates were activated by PMA at 80 nm and then incubated with PNS@Res or PNR@Res at the Res concentration of 20 µm for 12 h. RAW264.7 were pretreated with LPS (1 µg mL^−1^) for 12 h. Afterward, these drug‐incorporated PLTs were co‐cultured with activated RAW264.7. After incubation for 12 h, cells were washed and fixed. Dehydration process was performed using a graded ethanol series, and cell samples were subjected to SEM observation to identify the cell adhesion. In parallel, RAW264.7 was stained using DAPI, and PLTs were labeled using anti‐CD62p antibody. The cell adhesion was also observed using a fluorescence microscope. After treatment, the level of TNF‐α and IFN‐γ in supernatant of cell cultures was detected using ELISA kits. The ROS level of RAW264.7 after incubating with drug‐incorporated PLTs was measured using the fluorescent probe DCFH‐DA. In addition, the phenotype of RAW264.7 was also detected using a fluorescence microscope by staining RAW264.7 with anti‐CD86 antibody (Affinity Biosciences).

### The Interaction of PNR@Res‐Treated PLTs with Vascular Network

HUVEC were seeded in Matrix Gel for 6 h to allow for the formation of 3D vascular network.^[^ ^39^ ^]^ After the vascular network was fully established, these drugs‐incorporated PLTs were then co‐incubated with them for 12 h in the presence of LPS (1 µg mL^−1^) to mimic the inflammatory state. After treatment, the vascular networks were stained using DAPI. The numbers of formed tubes and junctions were calculated using the Image J based on the fluorescence images. The ROS level of HUVEC tubes after incubating with drug‐incorporated PLTs was verified via the fluorescent ROS probe DCFH‐DA. The cell adhesion of drug‐incorporated PLTs with the vascular networks was determined using immunofluorescent staining. Briefly, HUVEC tubes were stained using DAPI, and PLTs were labeled using anti‐CD62p antibody. The co‐localization of HUVEC tubes and PLTs was observed via a fluorescence microscope.

### In Vivo Targeting Efficiency and Biodistribution

Sprague‐Dawley (SD) rats were anesthetized, and then 50 µL of complete Freund adjuvant was subcutaneously injected into the hind paws to establish an adjuvant‐induced arthritis (AIA) rat model.^[^ ^40^ ^]^ To compare the in vivo uptake efficiency of PNS and PNR by circulating PLTs, AIA Rats were intravenously injected with 1 mL of PNS@C6 or PNR@C6 at the C6 content of 10 µg. At 2, 6, and 12 h post‐injection, PLTs were collected and the fluorescence intensity was analyzed using a flow cytometry.

To study the in vivo biodistribution of PNS and PNR in AIA rats, fluorescent dye DiR was loaded into the nanoparticles. The prepared PNS@DiR and PNR@DiR at DiR dose of 10 µg per rat were intravenously administered into AIA rats. The in vivo fluorescence imaging of arthritic joints at 2, 6, and 12 h post‐injection was obtained via an in vivo imaging system (IVIS, Perkin Elmer, USA). At the same time, arthritic joints and main organs were dissected, and the fluorescence signal was also measured using ex vivo imaging. The arthritic synovium was collected, embedded, and cut into thick sections, which were stained with DAPI and then observed using a fluorescence microscope.

### In Vivo Therapeutic Efficacy

AIA rats were divided into four groups and intravenously administered one of the following: PBS, Res, PNS@Res, or PNR@Res, at the Res dose of 1 mg kg^−1^ every other day for consecutive 14 days. During the treatment process, body weight, paw thickness, and joint score of all AIA rats were monitored. After treatment, rats were sacrificed, and ankle joints were dissected. Joint synovium was homogenized in cold PBS, and the level of TNF‐α and IL‐17A in homogenates was measured using corresponding ELISA kits. Ankle joints were fixed, decalcified, and embedded in paraffin. Sections were stained with H&E and observed using a fluorescence microscope. The expression of CXCL7 (PLT activation‐related biomarker) and CD86 (M1 type macrophages) in synovial tissues was determined using immunofluorescence staining. After treatment, rats were euthanized and blood was collected. White blood cells (WBCs), red blood cells (RBCs), and PLTs count in blood were detected using an automated hematology analyzer (Mindray, China). Also, serum levels of ALT, AST, and BUN were measured using corresponding assay kits (Nanjing Jiancheng, China).

### Statistical Analysis

All experimental results were analyzed using GraphPad Prism 8.0 (La Jolla, USA) and expressed in mean and standard error (SEM). Analysis of variance (ANOVA) and Tukey's test post hoc analysis were used to determine the differences between multiple experimental groups. A *p*‐value of less than 0.05 was considered statistically significant.

## Conflict of Interest

The authors declare no conflict of interest

## Supporting information



Supporting Information

## Data Availability

The data that support the findings of this study are available from the corresponding author upon reasonable request.
