# Supplementary material for: Rod‐Shaped Nanotherapeutics Alleviate Rheumatoid Arthritis by Precisely Disrupting Platelet‐Mediated Pathological Crosstalk via a Morphology‐Dependent Manner
Source: Adv Sci (Weinh). 2025 Nov 20;13(8):e18005. doi: 10.1002/advs.202518005 (PMC12884817; doi:10.1002/advs.202518005)
Supplement: Supplementary file 1 — Supporting Information [file ADVS-13-e18005-s001.docx]

Supporting Information

**Rod-Shaped Nanotherapeutics Alleviate Rheumatoid Arthritis by Precisely Disrupting Platelet-Mediated Pathological Crosstalk in a Morphology-Dependent Manner**

*Bin Zhang, Yuanyi Hua, Xin Wen, Zhumei Liao, Jianheng Ren, Yajun Weng*, Qin Wang**

Institute of Biomedical Engineering, College of Medicine, Southwest Jiaotong University, Chengdu, China

Email: Qin Wang: wangqin666@swjtu.edu.cn


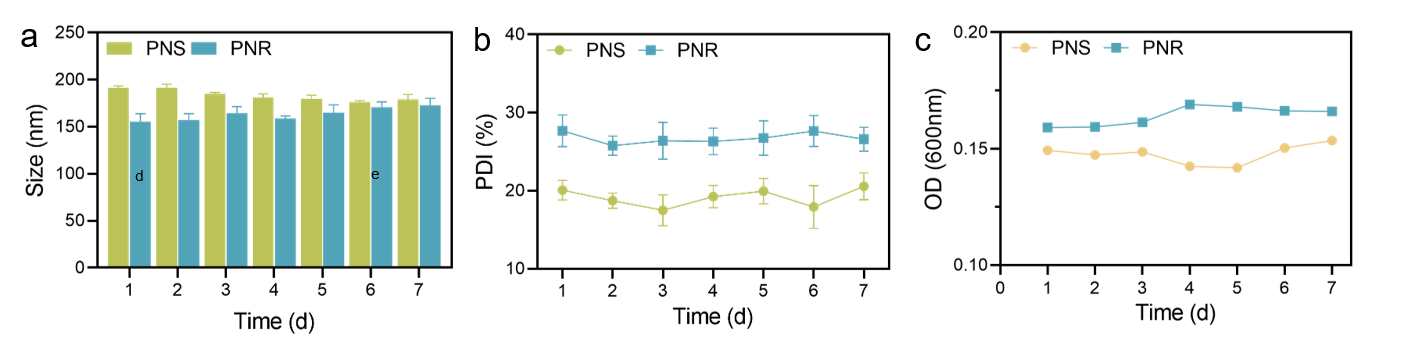


Figure S1. The changes in (a) particle size and (b) PDI of PNS and PNR after being stored in PBS for one week. (c) The turbidity of PNS and PNR when incubated with serum for a week. Results were shown as mean ± SD, *n*= 4.


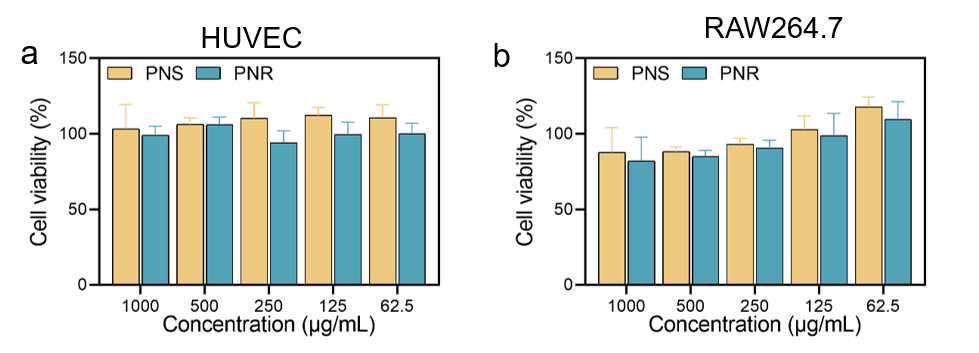


Figure S2. The cell viability of PNS and PNR in (a) HUVEC and (b) RAW264.7. Results were shown as mean ± SD, *n*= 4.


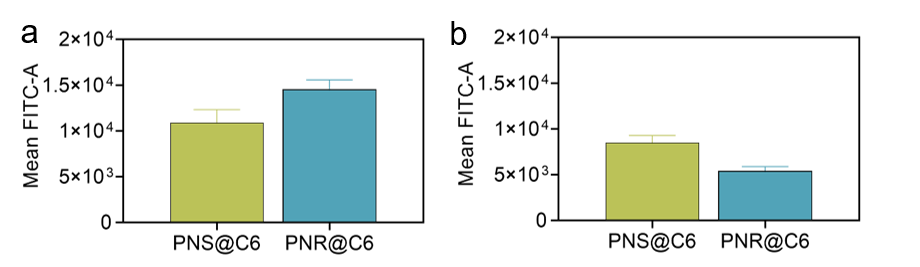


Figure S3. The (a) intracellular and (b) surface content of PNS@C6 and PNR@C6 after cellular uptake. Results were presented as means ± SD, *n*= 3.


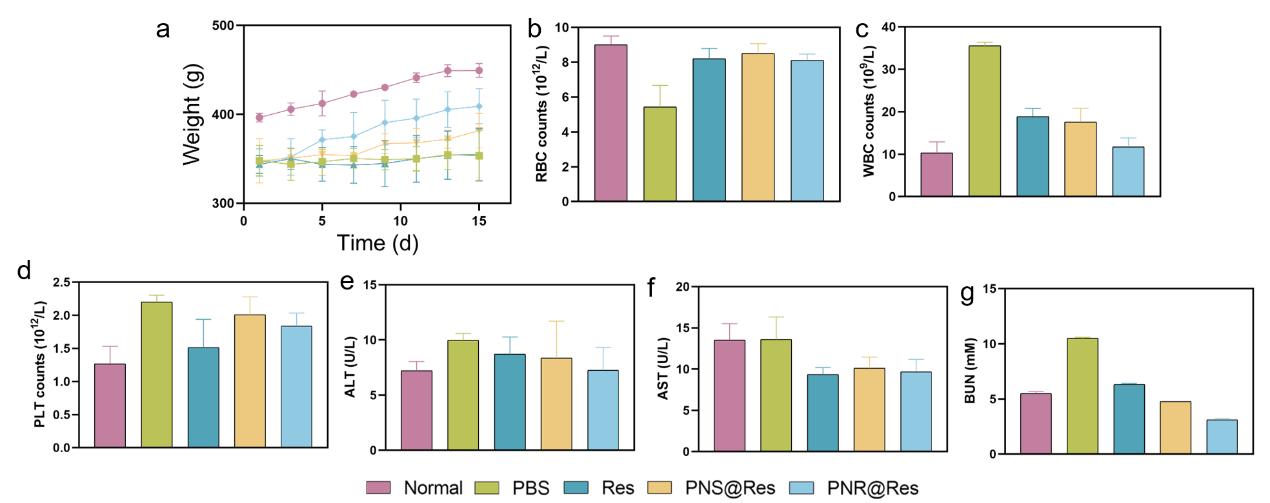


Figure S4. (a) The body weight changes during the treatment process. (b) RBC, (c) WBC and (d) PLT counts in blood after various treatments. The levels of (e) ALT, (f) AST and (g) BUN in serum after various treatment. Results were shown as mean ± SD, *n* = 6.


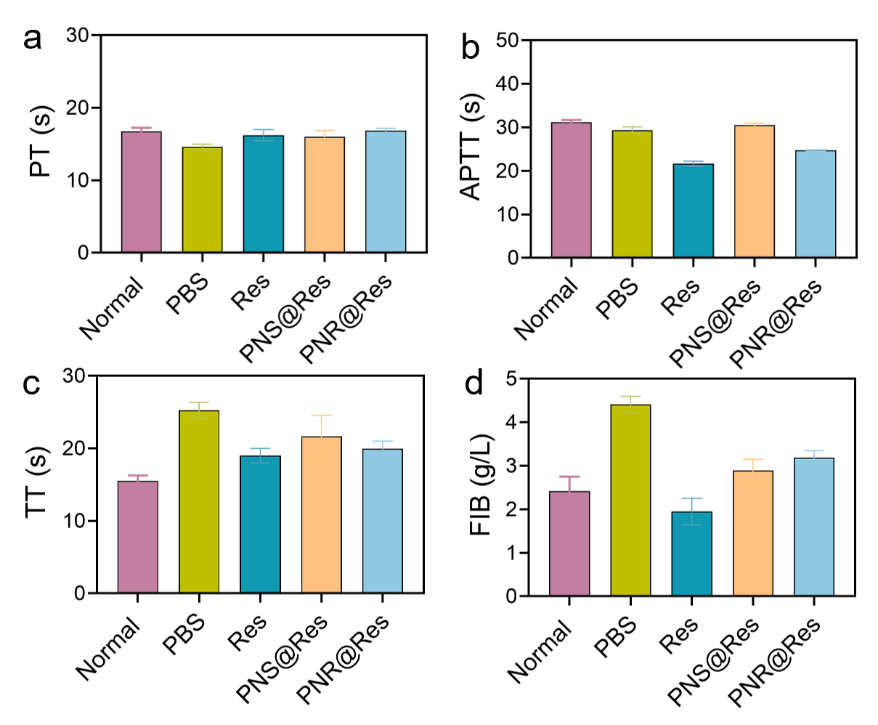


Figure S5. The coagulation indicators after treatment including (a) prothrombin time (PT), (b) activated partial thromboplastin time (APTT), (c) thrombin time (TT) and (d) fibrinogen (FIB). Results were shown as mean ± SD, *n* = 4.
